# Associations between Bisphenol A Exposure and Reproductive Hormones among Female Workers

**DOI:** 10.3390/ijerph121013240

**Published:** 2015-10-22

**Authors:** Maohua Miao, Wei Yuan, Fen Yang, Hong Liang, Zhijun Zhou, Runsheng Li, Ersheng Gao, De-Kun Li

**Affiliations:** 1 Key Laboratory of Contraceptive Drugs and Devices of NPFPC, Shanghai Institute of Planned Parenthood Research, WHO Collaborating Center for Research in Human Reproduction, Shanghai 200237, China; E-Mails: miaomaohua@163.com (M.M.); yangfen19880719@163.com (F.Y.); lucylhcn@163.com (H.L.); runshengli2007@gmail.com (R.L.); ersheng_gao@163.com (E.G.); 2 School of Public Health, Key Laboratory for Public Health Safety, WHO Collaborating Center for Occupational Health, Fudan University, Shanghai 200032, China; E-Mail: zjzhou@fudan.edu.cn; 3 Division of Research, Kaiser Foundation Research Institute, Kaiser Permanente Northern California, 2000 Broadway, Oakland, CA 94612, USA; E-Mail: de-kun.li@kp.org; 4 Department of Health Research and Policy, School of Medicine, Stanford University, Stanford, CA 94305, USA

**Keywords:** Bisphenol A, female hormones, endocrine disruptors

## Abstract

The associations between Bisphenol-A (BPA) exposure and reproductive hormone levels among women are unclear. A cross-sectional study was conducted among female workers from BPA-exposed and unexposed factories in China. Women’s blood samples were collected for assay of follicle-stimulating hormone (FSH), luteinizing hormone (LH), 17β-Estradiol (E2), prolactin (PRL), and progesterone (PROG). Their urine samples were collected for BPA measurement. In the exposed group, time weighted average exposure to BPA for an 8-h shift (TWA8), a measure incorporating historic exposure level, was generated based on personal air sampling. Multiple linear regression analyses were used to examine linear associations between urine BPA concentration and reproductive hormones after controlling for potential confounders. A total of 106 exposed and 250 unexposed female workers were included in this study. A significant positive association between increased urine BPA concentration and higher PRL and PROG levels were observed. Similar associations were observed after the analysis was carried out separately among the exposed and unexposed workers. In addition, a positive association between urine BPA and E2 was observed among exposed workers with borderline significance, while a statistically significant inverse association between urine BPA and FSH was observed among unexposed group. The results suggest that BPA exposure may lead to alterations in female reproductive hormone levels.

## 1. Introduction

Endocrine disrupting chemicals (EDCs) are a cluster of chemicals which can affect the endocrine system, including effects on hormone synthesis, secretion, or metabolism in the body. One such chemical, Bisphenol A (BPA), has brought about more and more concerns due to its wide spread exposure and potential harmful effects to human health [1,2]. BPA is a constitute of polycarbonate and epoxy resins, which may be used as the lacquer lining of food and beverage cans, and some dental sealants and composites. As a result of exposure from dietary and other sources, most people have BPA detected in their urine, despite their various lifestyles [2].

Accumulating literature documents the alterations of circulating reproductive hormone concentrations following BPA exposures in animal models [3,4,5]. Particularly, these effects can be observed at environmentally relevant low dose [6]. In female rodents, effects of BPA prenatal exposure on the oocyte, developing reproductive tract, and timing of sexual maturation were observed [7,8,9]. Similarly, BPA has been linked to several endocrine disorders including precocious puberty, hormone dependent tumors such as breast and prostate cancer, and several metabolic disorders including obesity, diabetes, and polycystic ovary syndrome in human studies [2].

Human studies demonstrating BPA’s effects on circulating levels of reproductive hormones were limited and inconclusive, particularly for women [10,11,12,13,14,15,16]. A significant and positive relationship was reported between BPA exposure and circulating androgen concentrations in a small study of 26 normal women and 47 women with ovarian dysfunction [17]*.*While no association was found between BPA and testosterone and E2 in a population-based prospective study [10]. An association between BPA and miscarriage have also been reported, indicating BPA’s possible disturbance to hormone homeostasis [18,19]. 

In the present study, we examined the associations between BPA level and concentrations of serum reproductive hormones including follicle-stimulating hormone (FSH), luteinizing hormone (LH), 17β-Estradiol (E2), prolactin (PRL), and progesterone (PROG) among female workers who were exposed to relatively high BPA in the workplace presently and cumulatively.

## 2. Experimental Section

### 2.1. Study Population

The population for this study was identified from participants of a study evaluating the health effects of BPA in workplace. The study design and the findings on male workers have been described elsewhere [20,21,22]. In brief, a retrospective cohort of BPA-exposed and un-exposed female workers were recruited. The exposed female workers came from manufacturers of epoxy resin in China, while the unexposed workers were from factories including garment factories, a textile factory, and a trade and commerce firm from the same jurisdiction of the health department overseeing the participating BPA-exposed factories. The study was approved by the committees for protection of human subjects from all participating institutes. All the workers in the study factories were invited to participate in the study and were asked to provide urine and blood samples after written informed consent forms were obtained. In the original cohort, a total of 431 female workers were recruited, the participation rate was 77.85% and 60.27% for exposed and unexposed groups, respectively. Female workers were eligible for the present study if they: (1) have been working in the current factory for more than six months; (2) were aged 18–45 years; (3) did not have major disease including diabetes, coronary heart disease, and chronic infections (e.g., tuberculosis). A total of 356 women were included in the present analysis, including 106 in exposed group and 250 in unexposed group. 

### 2.2. In Person Interview

Information on demographic characteristics, menstrual cycle, history of contraceptive methods usage and sexually transmitted diseases, as well as working history and lifestyle factors (smoking, alcohol, and caffeine consumption) were obtained by an in-person interview. 

### 2.3. Measurement of BPA Exposure

#### 2.3.1. TWA8 among Exposed Workers

In the exposed group, personal air sample monitoring was carried out for each workplace (workers in the same place were considered to have shared the similar exposure). We used the ESCORT ELF sampling pump with an inhalable fraction sampling head placed near the workers’ inhalation level. As described previously [23], the BPA exposure level during an 8-h shift was calculated using the Time-weighted Average (TWA8) for each individual who carried out the personal sample monitoring according to the following formula: TWA8 = ( airborne BPA concentrations × working hous)/8. TWA8 for a given BPA-exposed subgroup was the average of all individual TWA8s at this workplace.

#### 2.3.2. Urine BPA Measurement

All urine samples were collected using plastic tubes which were BPA-free. A single spot urine sample was collected from each unexposed female. For the exposed workers, one pre-shift and one post-shift urine sample was collected for each participating worker at the beginning and end of her working shift. To produce more stable measurements, we used the average of BPA levels from those two specimens among exposed workers who have provided both preshift and postshift urine samples. Seven women provided only pre-shift and 14 women provided only post-shift sample; thus only one urine sample was included for those seven participants. Total (free and conjugated) urine concentrations of BPA were examined through high performance liquid chromatography (HPLC). Briefly, urine samples were mixed with phosphorous acid buffer and b-glucuronidase (Sigma Chemical Co., St Louis, MO, USA) for hydrolyzation. Afterwards, samples were extracted twice with ether (HPLC grade; Dikma, Lake Forest, California) and supernatants were evaporated with nitrogen gas. The residue was dissolved in 60% acetonitrile and analyzed by HPLC with fluorescence detection. The assay was conducted by coauthors at the Department of Occupational Health and Toxicology, School of Public Health & WHO Collaborating Center for Occupational Health, Fudan University, Shanghai, China. To adjust for urine volume, we used creatinine-corrected (mg/g creatinine) BPA concentration in the analyses. Blank sample were randomly included during the assay and a sample with pre-defined concentration of 4.5 mg/L was included in parallel analysis and the correct reading of its concentration indicate no error occur during the assay. The limit of detection (LOD) was <0.31 μg/L BPA. Samples below the LOD were coded as LOD divided by the square root of 2, based on a conventionally accepted practice [24].

### 2.4. Serum Hormones Analysis

Venous blood samples were drawn from 8 a.m. to 10 a.m., and the serum was separated and frozen at −80 ℃ within eight hours. All samples were shipped to the laboratory in Shanxi Medical University on dry ice and analyzed for hormone concentrations. We assayed serum FSH, LH, E2, PRL, and PROG through a commercially available radioimmunoassay (RIA) kits provided by China Diagnostics Medical Corporations (Beijing, China). 

### 2.5. Statistical Analysis

Data analysis was performed using SAS version 9.1 (SAS Institute Inc., Cary, NC, USA). Descriptive statistics of demographic characteristics as well as urine BPA concentration for exposed and unexposed groups were obtained. The level of serum hormones and urine BPA concentrations were log-transformed to normalize the skewed distribution. FSH, LH, E2, PRL, and PROG levels were compared between exposed (higher and lower TWA8) and unexposed groups, as well as by categorized urine BPA level. To examine the dose response relationship we generated a new variable with value of 1 (for unexposed workers), 2 (Twa8 < 5), and 3 (Twa8 ≥ 5), which was then included in linear regression model with log-transformed FSH, LH, E2, PRL, and PROG as dependent variable, respectively.

Multiple linear regression analysis was also used to examine associations between urine BPA level (continuous) and reproductive hormones. The following factors were adjusted for: age, passive smoking, menstrual regularity (yes/no) and study site. Menstrual phases were further adjusted in a separate model using dummy variable after categorized by cycle day(cd) 1–7 (early follicular phase), cd 8–13 (late follicular phase), cd 14–15 (Ovulation), and cd 16–32 (Luteal phase) [25]. We stratified the analysis based on exposure status (exposed *vs.* unexposed workers) to examine whether the association differed across the two groups. We also performed sensitive analyses among: (1) those with a longer working year to examine whether the effects were cumulative; (2) those who did not change the job title in the current factory, a sub-sample of workers whose current BPA exposure level represented relatively well of the past exposure; and (3) those who did not use contraceptive medicine to reduce the impact of exogenous hormones. 

## 3. Results and Discussion

### 3.1. Results

Table 1 shows the participants’ demographic characteristics and urine BPA concentrations (after creatinine adjustment). The exposed and unexposed workers were comparable with regard to sites, age, marriage status, menstrual regularity, passive smoking exposure, and contraceptive methods, while educational level were lower for workers in the unexposed group(*p* < 0.05). The average age at menarche and years of working in the present factory were also similar across the two groups. As expected, the geometric mean of urine BPA concentration was higher in the exposed workers (22.2 μg/g) than unexposed workers (0.9 μg/g), and corresponding detection rate was 88.9% and 45.2%, respectively. Less than 2% of the participants reported regular smoking or alcohol consuming (data not shown).

**Table 1:** Characteristic of women in exposed and unexposed group.

Characteristics	Categories	Exposed (*n* = 106)	Unexposed (*n* = 250)	*p*
Site	Wuxi	19 (17.9)	42 (16.8)	0.1
	Yueyang	58 (54.7)	111 (44.4)	
	Yixing	29 (27.4)	97 (38.8)	
Education	Middle schoool and below	22 (20.8)	120 (48.0)	<0.0001
	High school	66 (62.3)	96 (38.4)	
	College and above	18 (16.9)	34 (13.6)	
Age (Years)	18–30	38 (35.9)	86 (34.4)	0.9
	31–40	52 (49.1)	129 (51.6)	
	41–45	16 (15.0)	35 (14.0)	
Marriage	Unmarried	8 (7.6)	17 (6.8)	0.5
	In marriage	95 (89.6)	218 (87.2)	
	Divorced or widowed	3 (2.8)	15 (6.0)	
Menstrual cycle	Regular	94 (88.7)	217 (88.6)	1.0
	Not regular	12 (11.3)	28 (11.4)	
Passive smoking	Yes	47 (44.3)	118 (47.4)	0.6
	No	59 (55.7)	131 (52.6)	
Current contraceptive methods usage	None	6 (5.9)	16 (6.7)	0.4
	Pills	7 (6.9)	7 (2.9)	
	IUD	60 (59.4)	137 (57.1)	
	Condom	27 (26.7)	79 (32.9)	
	Others	1 (1.1)	1 (0.4)	
Menarche age *		14.1 ± 1.5	14.2 ± 1.8	0.4
Year of working *		8.5 ± 6.8	9.5 ± 6.5	0.2
BPA	Detection rate	88.9	45.2	<0.0001
	geometric mean(95% CI) (ug/g cr)	22.2	0.9	<0.0001
		(12.4, 39.8)	(0.7, 1.1)	

* mean + std.

Table 2 shows the average levels of log-transformed hormone levels across different BPA exposure categories. Women with higher TWA8 had lower LH and higher E2 levels. We also observed non-significant trends of higher TWA8 BPA exposure associated with higher PRL and PROG levels. Comparison across urine BPA categories also showed a trend of higher PRL and PROG for women in the higher urine BPA categories, while only the association with PRL reached statistical significance. 

**Table 2:** Average hormone levels by BPA exposure (mean ± std, log-transformed).

BPA Exposure	FSH (mIU/mL)	LH (mIU/mL)	E2 (pg/mL)	PRL (ng/mL)	PROG (ng/mL)
By occupational exposure history					
Unexposed (*n* = 250)	1.05 ± 0.83	1.56 ± 0.98	3.71 ± 0.50	2.67 ± 0.68	1.00 ± 1.99
Exposed					
Twa8 < 5 (*n* = 61)	1.22 ± 0.78	1.75 ± 0.93	3.50 ± 0.43	2.70 ± 0.72	0.96 ± 1.86
Twa8 >= 5 (*n* = 45)	1.05 ± 0.62	1.27 ± 0.84 *	3.87 ± 0.41 *	2.74 ± 0.62	1.51 ± 1.87
By urine BPA level					
<limit of detection (LOD) (*n* = 146)	1.20 ± 0.70	1.57 ± 0.99	3.68 ± 0.45	2.59 ± 0.63 **^#^**	0.78 ± 2.01
LOD-75th (*n* = 103)	0.97 ± 0.88	1.58 ± 0.97	3.64 ± 0.56	2.63 ± 0.74	1.06 ± 1.78
>=75th (*n* = 82)	1.02 ± 0.88	1.60 ± 0.96	3.75 ± 0.47	2.85 ± 0.68	1.36 ± 2.06

* Comparison of hormones across occupational exposure (higher TWA8, lower TWA8, unexposed), ANOVA, *p* < 0.05; **^#^**, comparison of hormones between women with higher and lower urinary BPA, ANOVA, *p* < 0.05.

**Table 3:** Associations between urine BPA and female hormones *.

Hormones	Model 1		Model 2	
	Coefficient	*p*	Coefficient	*p*
**All participants**				
FSH	−0.03	0.17	−0.01	0.47
LH	−0.006	0.79	−0.005	0.81
E2	0.02	0.18	0.01	0.20
PRL	0.04	0.009	0.04	0.02
PROG	0.15	0.002	0.11	0.01
**Among exposed group**				
FSH	−0.03	0.38	−0.03	0.38
LH	0.04	0.42	0.05	0.28
E2	0.04	0.06	0.04	0.05
PRL	0.06	0.07	0.07	0.05
PROG	0.20	0.04	0.19	0.03
**Among unexposed group**				
FSH	−0.10	0.001	−0.07	0.006
LH	−0.03	0.44	−0.03	0.39
E2	0.02	0.18	0.02	0.21
PRL	0.05	0.05	0.04	0.11
PROG	0.21	0.004	0.15	0.02

* linear regression; model 1: adjusted for age, passive smoking, and study center; model 2: adjusted for age, passive smoking, study center and menstrual phase (dummy variables).

Table 3 shows associations between urine BPA concentrations (creatinine adjusted) and serum hormones using multivariable linear regression models. After adjusting for age, smoking status, menstrual regularity and study center, significant positive associations were observed between urine BPA concentration and PRL and PROG level, which are consistent with the findings in Table 2. Further adjustment for menstrual phase did not change the results noticeably. Similar associations were observed after the analyses were stratified by exposure status (exposed *vs.* unexposed workers), although the association between urine BPA and PRL was no longer statistically significant due to reduced sample size (*p* = 0.05 in the exposed group and *p* = 0.11 in the unexposed group). A positive association between urine BPA and E2 was observed among exposed workers with borderline significance (*p* = 0.05), while an inverse association between urine BPA and FSH was observed among unexposed group (*p* < 0.05).

We repeated the above analysis among those with longer working year, those who had never change the job title in the current factory, and those who did not use contraceptive medicine currently, similar results were observed. 

### 3.2. Discussion

The present study examined the relationship between BPA exposure and several serum reproductive hormones. A significant positive association was found between urine BPA level and serum PRL and PROG concentration. In addition, a positive association between urine BPA and E2 was observed with borderline significance among women in BPA-exposed group, while an inverse association between urine BPA and FSH was observed among unexposed group.

To our knowledge, this is the first time that BPA exposure is found to be linked to higher PRL level among adult females. However, this finding is consistent with reported findings from *in vitro* and *in vivo* studies. *In vitro* studies showed that except for estrogen receptors alpha, beta, gamma, BPA can also bind to membrane estrogen receptor (mER), and these membrane-bound receptors are capable of nongenomic steroid actions [3]. GH3/B6 pituitary cells, which express mER, respond to low level BPA exposure (in the picomolar to nanomolar range) by producing a calcium flux which leads to PRL release [26]. BPA can also induce prolactin gene expression and cell proliferation in both primary anterior pituitary cells and GH3 cells [27]. In an animal study, injecting approximately 15 mg/(kg·day) of BPA into neonatal Fisher 344 rat pups resulted in an increase in serum PRL levels [28]. Similarly, treatment of ovariectomized Wistar rats with BPA at doses of 11–250 mg/kg per day induced hyperprolactinemia [29]. The observed association between BPA and increased PROG in the present study is also supported by the finding of an alteration of PR expression following BPA exposure in nonhuman primates [30]. Similarly, the association between urine BPA and E2 among exposed workers is consistent with an animal study [27]. Thus, it is likely that high BPA exposure in women may alter the reproductive hormones of female. However, the associations between BPA and FSH and LH were inconsistent across different comparisons and no conclusion can be made. 

The unexposed group in our study also had detectable BPA. It is well recognized that ingestion is the most frequent route of BPA exposure in non-occupationally exposed population, while the exposed population may be exposed to BPA from additional routes of dermal, inhalation, and ingestion through exposure in the workplace [2]. Despite this difference, we found similar association with regard to PRL and PROG among exposed and unexposed women. Compared with unexposed group, we did not find a stronger association between BPA and PRL and progesterone among exposed group, although workers in the group had much higher BPA level. This indicated that lower dose BPA exposure may produce similar endocrine disrupting effects as suggested by previous studies [16].

BPA levels in the unexposed group in the present study are lower than those that were reported in other countries, as well as in a study of school-aged students conducted by our research team in Shanghai, China in 2011 [31]. Consuming heavily packaged food was reported to be a major source of exposure of BPA, while in most Chinese rural areas, fresh cooked foods were the most common food type [32]. This lifestyle pattern may explain the low BPA levels in the study population from the unexposed group given that they came from relatively rural or small town.

Our study has several limitations: (1) we did not restrict the menstrual phases when recruiting women. The reproductive hormones we examined are ovary-dependent and thus have large variation within a menstrual cycle, particularly for FSH, LH, and E2. This variation could potentially lead to non-differential misclassifications and reduce our ability to detect existing association, although we have controlled for menstrual phases in the analysis. (2) BPA is very rapidly metabolized, with an elimination rate of hours [33]. The spot urine samples (single in the un-exposed and twice in the exposed group) collected may reflect only very recent exposures and cannot characterize average BPA exposures for individuals. Thus, the non-differential misclassification of BPA exposure cannot be avoided and may bias the results toward null. (3) BMI was not collected in the present study and thus was not included in the regression analysis. BMI was reported to be associated with hormone levels like serum E2 [34,35]. As BPA may have obesity-promoting effects [36], BMI might be the intermediate variable on the pathway of BPA and hormones. Therefore, it may not be appropriate to adjust for BMI in our model.

## 4. Conclusions

We have reported that BPA exposure was associated with adverse reproductive endpoints among male workers who were occupationally exposed to BPA, including poor semen quality, decreased sexual function, and reproductive hormone and sperm methylation alteration [20,21,22,23]. The present study added to the evidences that BPA is also disruptive to women’s hormone homeostasis. Together, these data raised concerns about the potentially deleterious impact of BPA on human reproductive health. Public awareness of on these deleterious effects should be strengthened and protection against BPA exposure should be promoted. 

## Abbreviations

BPA, Bisphenol A; FSH, follicle-stimulating hormone; LH, luteinizing hormone; E2,17β-Estradiol; PRL, prolactin; PROG, progesterone; ANOVA, analysis of variance.
